# The vaginal microbiome of South African pregnant women living with human immunodeficiency virus (HIV) with and without *Chlamydia trachomatis* infection

**DOI:** 10.1186/s12905-024-03246-1

**Published:** 2024-07-18

**Authors:** Nonkululeko G. Mabaso, Bongekile Ngobese, Hamilton Ganesan, Donald van der Westhuizen, Wail M. Hassan, Nathlee S. Abbai

**Affiliations:** 1 School of Clinical Medicine Laboratory, College of Health Sciences, University of KwaZulu-Natal, Durban, South Africa; 2 Inqaba Biotechnical Industries (Pty) Ltd, Pretoria, South Africa; 3 Department of Biomedical Sciences, School of Medicine, University of Missouri-Kansas City, Kansas City, MO USA

**Keywords:** *Chlamydia trachomatis*, Vaginal microbiota, Pregnant women, HIV

## Abstract

**Background:**

*Chlamydia* genital infections continue to be a serious health concern globally. Previous studies have reported that *Chlamydia trachomatis* infection alters the vaginal microbiota of infected women. This study investigated differences in the vaginal microbiome of South African pregnant women living with HIV with and without *C. trachomatis* infection.

**Methods:**

This was a cross-sectional study among 385 pregnant women, recruited from the King Edward VIII Hospital in Durban, South Africa. *C. trachomatis* was detected using the Applied Biosystems™ TaqMan^®^ Assays. A total of 40 samples, 20 *C. trachomatis* positive and 20 *C. trachomatis* negative, were selected for sequencing. The sequencing of the vaginal microbiome was performed using the PacBio platform. Statistical analysis was performed on IBM SPSS version 26.

**Results:**

The prevalence of *C. trachomatis* infection was 12.2% (47/385). The genus *Gardnerella* (32.14% vs. 24.02%) and species in the genus *Gardnerella* (31.97% vs. 24.03%) were more abundant in the *C. trachomatis*-infected group compared to the uninfected group. *Lactobacillus iners* were also more abundant in the *C. trachomatis*-infected women (28.30%) compared to the uninfected women. However, these observed patterns did not reach statistical significance. Discriminant analysis showed that the class *Alpha-Proteobacteria*; order *Bacillales*; family *Enterococcaceae*; the genera *Enhydrobacter*, *Enterococcus*, and *Parabacteroides*; *Enterococcus* spp.; and *Pseudomonas stutzeri* significantly contributed to a model separating *C. trachomatis*-infected women from the uninfected group (*p* < 0.05).

**Conclusion:**

The organisms and taxa that significantly contributed to separating the vaginal microbiota of *C. trachomatis*-infected women from the uninfected women in this study cohort have not been previously observed in association with *C. trachomatis* infection or the vaginal microbiota. Future studies in larger cohorts that will investigate the role of these microorganisms in *C. trachomatis* infection and the vaginal microbiota are required.

## Introduction

The vaginal flora has been shown to have an important role in the homeostasis and health of the female reproductive tract [1]. The vaginal and cervical microbiomes consist of diverse microorganisms that co-exist in a dynamic equilibrium, creating intricate interactions with one another and with the host [2]. The *Lactobacillus* genus typically predominates the vaginal microbiota of healthy reproductive-age women [1–3]. Most healthy females display the dominance of one species among lactobacilli, such as *L. crispatus*, *L. iners*, *L. jensenii*, and *L. gasseri* [2]. *Lactobacillus* spp. maintain a healthy vaginal environment and prevent invasion of the genital tract by pathogenic microorganisms, including pathogens that cause sexually transmitted infections (STIs) [1, 2, 4]. *Lactobacillus* spp. protects the genital tract from invading pathogens by maintaining a low vaginal pH (< 4.5), secreting bacteriostatic and bactericidal compounds, and competing for nutrients with these pathogens [2, 5].

In addition to lactobacilli, other microorganisms inhabit the cervical and vaginal environment, including anaerobes such as *Prevotella* spp., *Gardnerella vaginalis*, and *Atopobium vaginae* [3]. The shift from a healthy *Lactobacillus* spp. dominant genital microbiota to a dysbiosis dominated by these anaerobes is defined as bacterial vaginosis (BV) [2, 3]. BV is one of the most prevalent gynecologic disorders in women of reproductive age worldwide [6]. BV has been associated with the increase in acquisition and transmission of STIs, including *C. trachomatis*, human papillomavirus (HPV), and human immunodeficiency virus (HIV) [1, 5]. Furthermore, an imbalance in the vaginal microbiota during pregnancy is associated with early and late miscarriage, an increased risk of post-abortal infections, premature rupture of membranes, postpartum endometritis, and preterm birth [6, 7].

*C. trachomatis* is an obligate intracellular bacterial pathogen that causes one of the most common curable STIs, chlamydia [2, 5]. Between 2010 and 2019, the global prevalence of *C. trachomatis* infection among pregnant women ranged from 1 to 36.8% [8]. In South Africa, research studies have shown that the prevalence of this infection among pregnant women is a staggering 20% [9] to 26% [10]. *C*. *trachomatis* infection is often asymptomatic in women [11] and untreated infections lead to abnormal vaginal discharge, dysuria, pelvic inflammatory disease (PID), ectopic pregnancy, and tubal infertility [5, 12]. Previous studies have demonstrated that women infected with *C. trachomatis* are more likely to have dysbiotic vaginal microbiota or *L. iners* predominance compared to uninfected women [4, 5]. *L*. *iners* has been shown to be present in both the normal vaginal microbiota and the dysbiotic vaginal microbiota associated with STIs and BV [7].

Previous studies have reported that *C. trachomatis* infection alters the vaginal microbiota of infected women. Assuming that alterations in the vaginal microbiome may affect susceptibility to *C. trachomatis*, investigating the composition of the vaginal microbiota associated with *C. trachomatis* infection is essential. Therefore, this study aimed to investigate differences in the vaginal microbiome of pregnant women living with HIV with and without *C. trachomatis* infection. We hypothesized that there are differences in the vaginal microbial profiles of women with and without *C. trachomatis* infection.

## Materials and methods

### Ethical statement

All ethical approvals were secured before study commencement.

### Study design and population

This was a sub-study of a larger cross-sectional study among pregnant women attending the antenatal clinic at the King Edward VIII Hospital in Durban, South Africa. For the larger study, the sample size was calculated based on the prevalence of HIV and STIs in our current setting. A total of 385 pregnant women were recruited between October 2020 and April 2021. At the clinic, women were educated on the complications of STIs during pregnancy and provided with information on risk reduction for STIs. Women were enrolled in this study if they were living with HIV, 18 years of age and older, pregnant, willing to provide written informed consent, willing to provide vaginal swab samples, and willing to provide sociodemographic, behavioral, and clinical data. The study participants provided their identity information, such as first names, surnames, and identity (ID) numbers or dates of birth. For data capture and analysis purposes, the study participants were assigned study ID numbers. For data capture from study participants, only a single structured questionnaire was administered. To reduce the bias in reporting of sexual behavior, women were encouraged to complete the details of sexual behavior by themselves. Each enrolled woman provided self-collected vaginal swabs (low-vaginal swabs) for detection of *C. trachomatis*. The women were provided with education and guidance on sample collection. They were instructed to insert the dry swab at least 2 cm into the vagina and gently swirl it around to collect the required sample. For the microbiome analysis, we selected samples that only had either *C. trachomatis* in them or no *C. trachomatis* with no other STI, which would act as a confounder. Since all women were living with HIV, HIV status was not a confounder in this analysis.

### Laboratory procedures

#### Sample processing and DNA extraction

The collected vaginal swabs were placed in 2 ml of phosphate-buffered saline (PBS). The solution was vortexed to dislodge the cells from the swabs, and the swab was discarded. The remaining suspension was centrifuged at 14,000 rpm for 10 min, and the supernatant was discarded. Recovered pellets were then subjected to DNA extraction using the PureLink Microbiome Kit (ThermoFisher Scientific, USA), according to the manufacturer’s instructions. The concentration of extracted DNA was determined using a NanoDrop spectrophotometer (ThermoFisher Scientific, USA). DNA samples were stored at -20 °C until further molecular analysis.

#### Detection of C. trachomatis

*C. trachomatis* was detected using the Applied Biosystems™ TaqMan^®^ Assay using commercially available primers and probes specific for *C. trachomatis* (Ba04646249_S1). The assay targets the translocated actin-recruiting phosphoprotein gene from this pathogen. Each PCR reaction was performed in a final volume of 20 µl comprising 1 µl FAM-labeled probe/primer mix, 5 µl Fast Start 4x probe master mix (ThermoFisher, Part No. 4,444,434), 2 µl template DNA, and 11 µl nuclease-free water. No-template and positive controls (TaqMan Vaginal Microbiota Extraction Control; cat no. A32039) were also included. PCR amplification was performed on the QuantStudio™ 5 Real-Time PCR detection system (ThermoFisher Scientific, USA), in a 96-well microtiter reaction plate. Amplification was performed at 95 °C for 30 s followed by 45 cycles comprising of denaturation at 95 °C for 3 s and annealing at 60 °C for 30 s. Detection of amplified fluorescent products was conducted at the end of the annealing phase. The raw fluorescent data that included the Ct mean values were automatically generated by the QuantStudio™ 5 Real-Time PCR system software.

#### Next generation sequencing

A first round PCR was performed using the modified (5’amino-PB M13 adaptor) universal full-length *16 S ribosomal* RNA primers forward primer: 27-F: /5AmMC6/GTAAAACGACGGCCAGT AGRGTTYGATYMTGGCTCAG and reverse primer: 1492-R: /5AmMC6/CAGGAAACAGCTATGAC RGYTACCTTGTTACGACTT. The resulting amplicons were then barcoded with PacBio M13 barcodes through PCR and confirmed with gel electrophoresis. The barcoded amplicons were then measured using Qubit dsDNA HS assay and normalized to 10 nM for pooling. An SMRTBell library preparation was performed on the pooled samples following PacBio SMRTBell Express Template Prep kit 2.0. Samples were demultiplexed and CCS reads were produced using the PacBio SMRTLINK v10.1. PacBio was performed to sequence the full length of the *16 S* amplicon at high quality. The sequencing depth was a minimum of 8000/sample.

### Statistical analysis

Model quality was evaluated using Wilks’ λ, a Chi-square test, canonical correlations, and eigenvalues. The Wilks’ λ statistic represents the amount of variance not explained by group membership, and therefore, this value approaches zero in models able to predict group membership with high fidelity [13]. The Chi-square test tests whether the model represents a significant improvement over the null model (i.e., a model with no variables) with *p* values < 0.05 indicating significance [13]. Although canonical correlations and eigenvalues are specific to each discriminant function, each of the discriminant models in this study contains a single discriminant function (the number of discriminant functions is equal to the number of groups minus one and we only had two groups, the *Chlamydia* positive and the uninfected groups), which means canonical correlations and eigenvalues in this sitting directly reflect on the quality of their respective models. Canonical correlation is the correlation between the discriminant function and group membership, which means higher correlations are found in stronger models [14]. The eigenvalue reflects the amount of variance explained by the discriminant function, and greater values are often found in high-quality models; the greater this value, the better the quality of the discriminant function [15]. The next question we addressed was whether a signature vaginal microbiome characteristic of *C. trachomatis* infection exists. To answer this question, we used discriminant analysis. Discriminant analysis was performed using the stepwise method, which only incorporates variables if they significantly improve the discriminant model. Discriminant analysis was performed using IBM SPSS version 26 (IBM Corporation, Armonk, NY). Characterization of microbiome alterations in chlamydia infection: Pairwise comparisons were performed using a two-tailed t-test with Holm-Sidak correction for multiple testing; both were performed using GraphPad Prism version 6 for Windows (GraphPad Software, San Diego, California USA, www.graphpad.com*).* Missing data was excluded from the analysis.

## Results

### Baseline characteristics and prevalence of *C. trachomatis* infection

A total of 385 pregnant women living with HIV were tested for *C. trachomatis* infection. The median (Q1-Q3) age of the study women was 30.0 years (25.0–36.0). A high proportion of the study women had completed high school (77.1%), were unemployed (75.1%) and were unmarried (87.3%). Of the 385 women, 47 (12.2%) tested positive for *C. trachomatis*. The median (Q1-Q3) age of the women who tested positive was 26.0 years (21.5–32.0). Among the women who tested positive for *C. trachomatis*, 89.4% had completed high school, 85.1% were unemployed, and 97.9% of the women were unmarried. An overview of study population demographics and clinical data has been published elsewhere [16]. Of the 47 samples that tested positive for *C. trachomatis* infection, only 20 had *C. trachomatis* infection with no co-infection. Therefore, 40 samples, 20 *C. trachomatis* positive and 20 *C. trachomatis* negative were selected for the vaginal microbiome analysis.

### Microbiome signature in C. trachomatis infection

Pairwise comparisons while correcting for multiple testing over the combined number of taxa i.e., the number of phyla, classes, orders, families, genera, and species totaled 722 taxa.

**Fig. 1:**
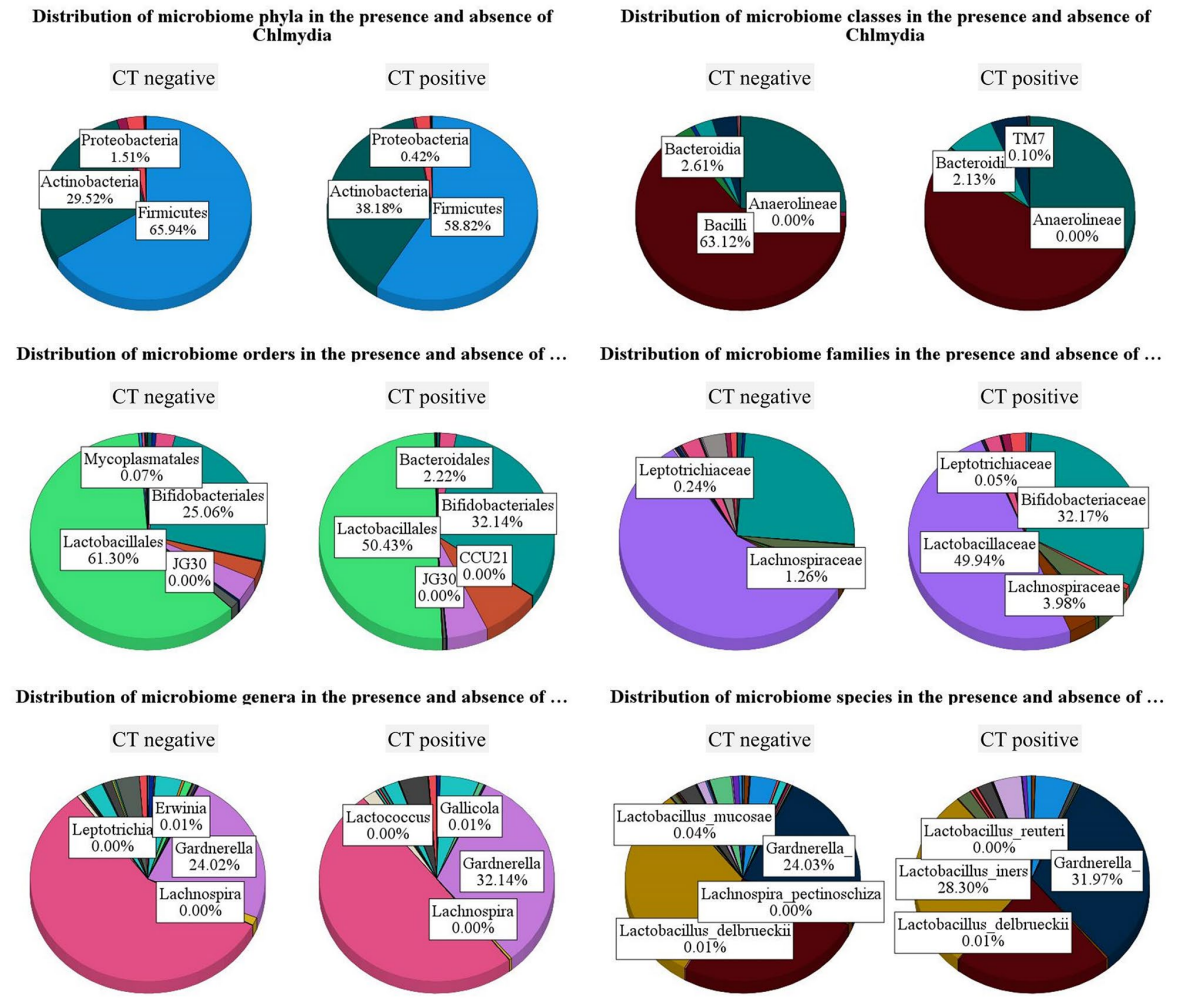
Microbiome alteration due to *C. trachomatis* (CT) infection

Firmicutes were predominant in the vaginal microbiota of both groups, with a higher abundance of 65.94% in the uninfected group compared to 58.82% in infected women. Actinobacteria were more abundant in *C. trachomatis*-infected women (38.18%) compared to 29.52% in the uninfected group. Proteobacteria was the least abundant in both groups, with a slightly higher abundance in the uninfected group (1.51% vs. 0.42% in the *C. trachomatis*-infected group). The analysis at the order level showed a higher abundance of Bifidobacteriales in the *C. trachomatis*-infected women (32.14% vs. 25.06% in the uninfected group). Lactobacilalles were more abundant in the uninfected group (61.30%) compared to 50.43% in the *C. trachomatis*-infected group. The family *Lachnospiraceae* was more abundant in the *C. trachomatis*-infected group (3.98%) compared to the uninfected group (1.26%). At the genus level, *Gardnerella* was more abundant in the *C. trachomatis*-infected group (32.14%) compared to the uninfected group (24.02%). Similarly, at the species level, the species annotated as *Gardnerella* were more abundant in the *C. trachomatis*-infected group (31.97%) compared to the uninfected group (24.03%). In addition, *L. iners* were also more abundant in the *C. trachomatis*-infected women (28.30%) compared to the uninfected women. Using the Holm-Sidak method there were no significant differences between women with *C. trachomatis* infection and the uninfected group (data not shown). The main taxa within each taxonomic rank (i.e., phylum, class, order, family, genus, and species) are shown with some visible, although not statistically significant, differences between infected and uninfected women (Fig. 1).

Discriminant analysis performed on each of the taxonomic ranks individually produced models of widely different qualities. However, the phylum data failed to produce a stepwise model in SPSS; therefore, we included all variables in the phylum model. In addition, we ran the stepwise discriminant analysis on the entire data set containing all taxa from all taxonomic ranks, as well as on the combined taxa included in the stepwise models. All models met statistical significance, except for the class model. Among the models representing single taxonomic ranks, the genus model was the strongest, with the lowest Wilks’ λ (0.045) and highest correlation (0.977) and eigenvalue (21.099). The very best model, however, was the one created from the combined taxa from all taxonomic ranks, showing a Wilks’ λ of 0.001, eigenvalue of 1052.44, and perfect correlation with group membership. The model created from the selected taxa of stepwise models did not overperform the genus model (Table 1).

**Table 1:** Properties of the vaginal microbiome discriminant model

Taxonomic ranks	Wilks’ λ	*p*-value	Eigenvalue	Canonical correlation
Phylum	0.425	0.047	1.353	0.758
Class	0.875	0.143	0.029	0.353
Order	0.603	0.001	0.657	0.630
Family	0.766	0.002	0.306	0.484
Genus	0.045	6.6 × 10^− 12^	21.099	0.977
Species	0.726	0.003	0.377	0.523
Selected	0.266	8.2 × 10^− 7^	2.757	0.857

Note: all models were constructed using the stepwise method, except for the phylum model, where all variables were forced into the model

**Fig. 2:**
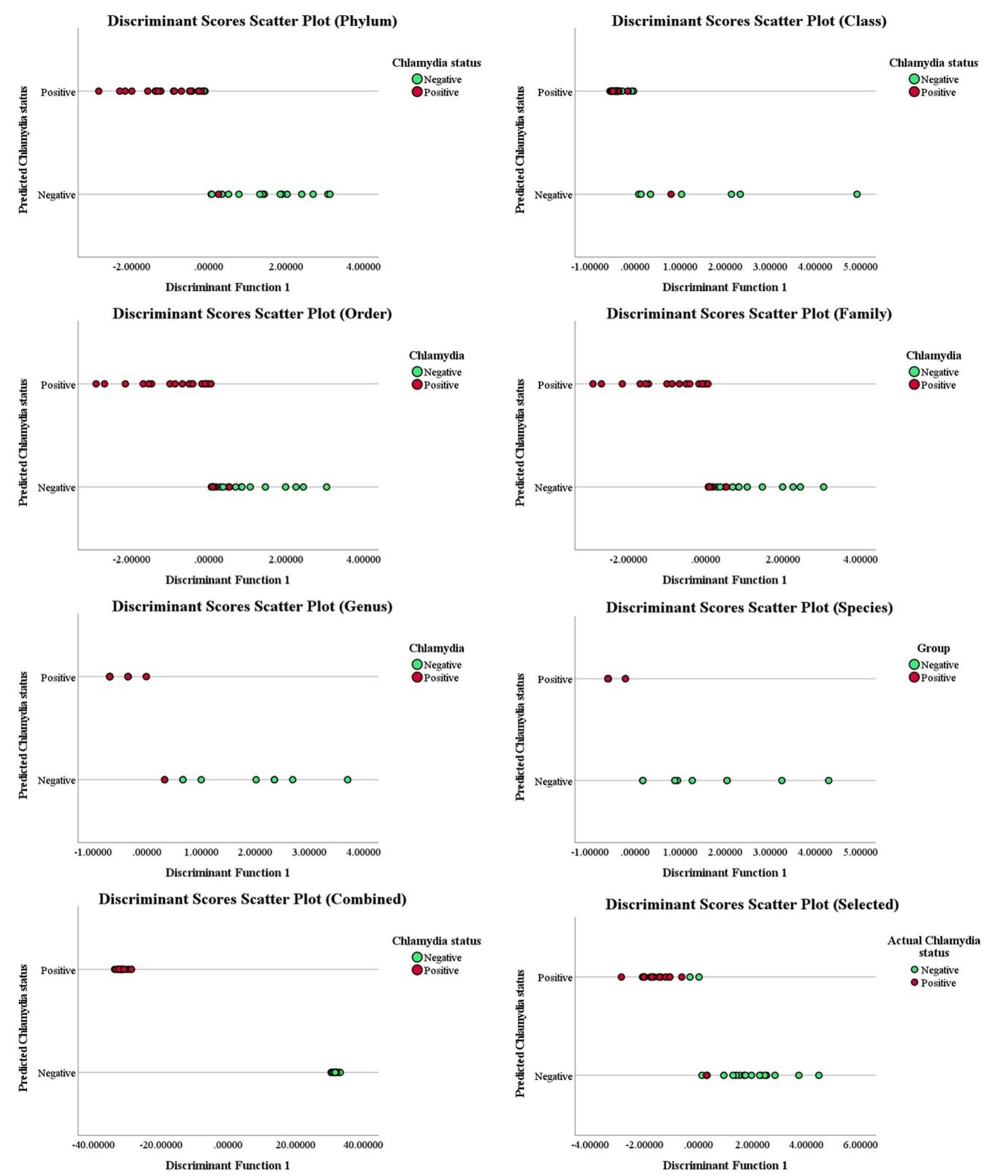
Score plots of the discriminant models

**Fig. 3:**
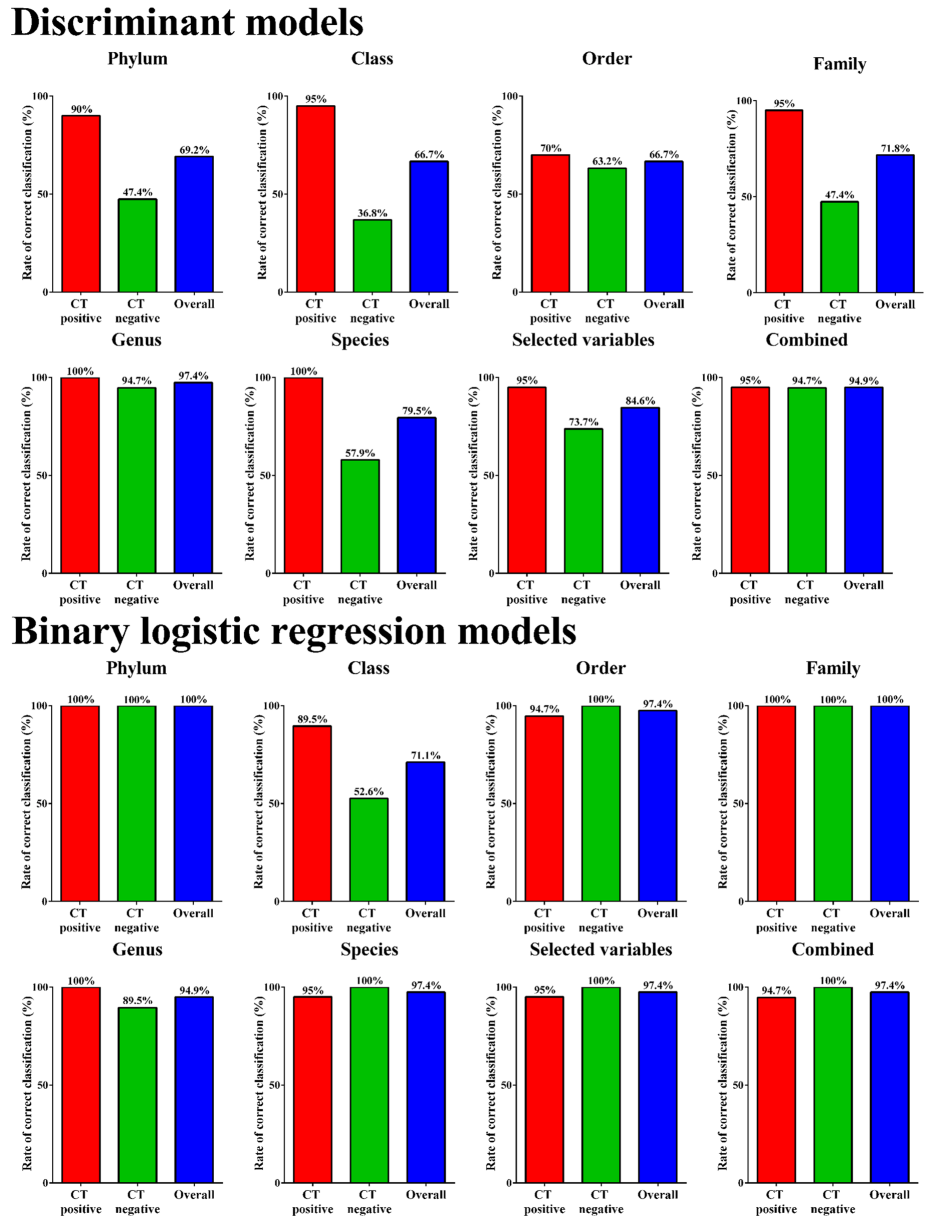
Fidelity of the discriminant models according to *Chlamydia* status. Data shown are based on stepwise models except for phylum data where a stepwise model could not be produced

Separation of *C. trachomatis* positive women from uninfected women on discriminant scores scatter plots was observed, although for genus and species models, multiple participants overlapped making it difficult to predict the rates of correct classification (RCC) of each model (Fig. 2). Therefore, we examined the RCCs calculated in SPSS. As expected, the highest RCCs were obtained using the genus and combined taxa models, with RCCs of 97% and 95%, respectively (Fig. 3).

Table 2 depicts the taxa included in each of the stepwise models with the corresponding measures of importance, the Wilks’ λ statistic, the standardized canonical discriminant function coefficient (SCDFC), and a significance *p*-value. Taxa of each model are listed in descending order of their corresponding SCDFCs. The discriminant analysis showed that the class: Alpha-Proteobacteria (*p =* 0.029); order: Bacillales (*p* = 0.018); family: *Enterococcaceae* (*p* = 0.002); genus: *Enhydrobacter* (*p* = 0.044), *Enterococcus* (*p* = 0.004) and *Parabacteroides* (*p* = 0.020); and species: *Enterococcus* spp. (*p* = 0.005) and *Pseudomonas stutzeri* (*p* = 0.009) were significantly associated with *C. trachomatis* infection.

**Table 2:** Vaginal microbiome alterations in *C. Trachomatis* infection

Taxonomic ranks	Discriminant Analysis significant taxa	Wilks’ λ	*p*-value	SCDFC
Class	*Alpha-Proteobacteria*	0.875	**0.029**	1.000
Order	*Mycoplasmatales* *Bacillales* *Clostridiales* *Pasteurellales*	0.930 0.858 0.921 0.979	0.104 **0.018** 0.082 0.383	0.762 0.749 −0.725 −0.584
Family	*Enterococcaceae*	0.766	**0.002**	1.000
Genus	*Collinsella* *Enhydrobacter* *Luteimonas* *Trabulsiella* *Enterococcus* *Chryseobacterium* *Brevibacterium* *Phascolarctobacterium* *Persicirhabdus* *Brachybacterium* *Raoultella* *Aerococcus* *Parabacteroides* *Escherichia* *Unknown*	0.912 0.894 0.912 0.949 0.801 0.926 0.916 0.959 0.972 0.972 0.943 0.960 0.863 1.000 1.000	0.067 **0.044** 0.067 0.167 **0.004** 0.095 0.073 0.215 0.311 0.311 0.144 0.220 **0.020** 0.955 0.957	2.634 2.584 2.084 2.05 2.005 −1.865 1.855 −1.824 1.54 1.19 1.076 0.867 0.73 −0.675 −0.578
Species	*Enterococcus* spp. *Pseudomonas stutzeri*	0.808 0.829	**0.005** **0.009**	0.685 0.618
Combined	*Sphingomonas asaccharolytica* *Collinsella aerofaciens* *Enterococcaceae* *Pediococcus* *Brevibacterium paucivorans* *Luteimonas* *Enhydrobacter* *CCU21* *Lactobacillus mucosae* *Tenericutes* *Enterococcus* *Actinobaculum* *Paucimonas* *Bergeriella* *Parvimonas* *Deinococci* *Dermabacteraceae* *Clostridium perfringens* *Granulicatella balaenopterae* *Sphaerochaetales* *Chlorophyta*	0.921 0.944 0.767 0.997 0.950 0.914 0.897 0.973 0.959 0.936 0.808 0.925 0.977 0.973 0.949 0.973 0.947 1.000 0.914 0.944 0.990	0.088 0.154 **0.002** 0.734 0.176 0.074 **0.049** 0.324 0.221 0.125 **0.006** 0.095 0.364 0.324 0.172 0.324 0.163 1.000 0.074 0.154 0.560	30.652 -19.046 15.468 10.926 10.526 10.001 9.076 8.772 8.666 7.405 -4.902 4.552 -3.535 3.258 -3.216 3.185 2.119 1.754 -1.501 -1.277 -1.130
Selected	*Enterococcaceae* *Enhydrobacter* *Luteimonas* *Chryseobacterium* *Persicirhabdus* *Collinsella* *Aerococcus* *Brevibacterium*	0.766 0.894 0.912 0.926 0.972 0.912 0.988 0.916	**0.002** **0.044** 0.067 0.095 0.311 0.067 0.507 0.073	1.238 1.053 0.999 −0.832 0.678 0.677 0.594 0.490

Note: *p*-values highlighted in bold indicate statistical significance

## Discussion

*Chlamydia* genital infections continue to be a serious health concern globally [17]. To our understanding, there is minimal data in South Africa on the differences in the vaginal microbiome of pregnant women living with HIV with and without *C. trachomatis* infection. This study aimed to fill this knowledge gap. In this study, the vaginal microbiome signature of *C. trachomatis* infection was assessed. We hypothesized that there are differences in the vaginal microbial profiles of women infected with *C. trachomatis* compared to uninfected women. Our hypothesis was accepted. From the study, we found that the vaginal microbial profiles of the women with *C. trachomatis* infection were different from those with no infection. Our analysis showed that *Enhydrobacter*, *Enterococcus*, *Parabacteroides*, *Enterococcus* spp., and *P. stutzeri* were associated with *C. trachomatis* infection.

In the current study, the main taxa within each taxonomic rank showed some visible, although not statistically significant, differences between *C. trachomatis*-infected and uninfected women. Despite the lack of statistically significant differences, discriminant analysis was able to separate the groups. The analysis of taxonomic classification at the phylum level showed that all samples were mainly composed of Firmicutes, Actinobacteria, and Proteobacteria, with differences in their abundances across the groups. Firmicutes were predominant in the vaginal microbiota of both groups, with a higher abundance in the uninfected women compared to the *C. trachomatis*-infected women. Similarly, a study conducted by Ceccarani et al. [2] showed that Firmicutes dominated the vaginal microbiota of all the study groups, with a higher abundance of 92.40% in healthy women compared to 89.80% in *C. trachomatis* subjects.

In contrast, Actinobacteria were more abundant in *C. trachomatis*-infected women (38.18%) compared to the uninfected group (29.52%). Previous studies have also reported similar observations. Ceccarani et al. [2] also observed a higher abundance of Actinobacteria in *C. trachomatis*-infected women (5.6%) compared to healthy women (3.4%). Similar to previous studies [3, 18], the Proteobacteria phylum was the least abundant in both groups, with a slightly higher abundance in the uninfected group. Raimondi et al. [18] also observed a lower abundance of Proteobacteria in *C. trachomatis*-infected women compared to uninfected women (1.7% vs. 4.8%).

The analysis of the vaginal microbiota composition at the order level showed a higher abundance of Bifidobariales in the *C. trachomatis*-infected women. The order Lactobacilalles had a higher abundance in the uninfected group. The family *Lachnospiraceae* was more abundant in the *C. trachomatis*-infected group (3.98%) compared to the uninfected group (1.26%). Consistent with our findings, Ceccarani et al. [2] also observed an increased abundance of *Lachnospiraceae* in *C. trachomatis-*infected women (8.10%) compared to healthy women (3.16%).

Previous studies have demonstrated that women infected with *C. trachomatis* are more likely to have a *Lactobacillus iners*-dominated vaginal microbiota compared to uninfected women [4, 5]. In addition to *Gardnerella* being more abundant in the *C. trachomatis*-infected women at the species level, *L. iners* was shown to be more abundant in this group compared to the uninfected women. Similar to our findings, previous studies have also reported a higher abundance of *L. iners* in *C. trachomatis*-infected women compared to healthy women [2–4]. A study conducted among Dutch women showed that women who had a vaginal microbiota dominated by *L*. *iners* were more at risk of acquiring *C*. *trachomatis* infection [19]. *L*. *iners* has been shown to be present in both the normal vaginal microbiota and the dysbiotic vaginal microbiota associated with STIs and BV [7]. Hence, *L. iners* is considered a transitional species, colonizing the vagina after alterations in the vaginal microbiota [2].

There is a paucity of data on the association of *Enhydrobacter*, *Enterococcus*, *Parabacteroides*, *Enterococcus* spp., and *P. stutzeri* with *C. trachomatis* infection and the vaginal microbiota. *Enterococcus* spp. are Gram-positive, facultative anaerobic microorganisms [20]. These organisms are part of the gastrointestinal tract commensal flora; however, they may also be opportunistic pathogens [20, 21]. In addition, they may colonize the female reproductive tract, and vaginal colonization increases in females with aerobic vaginitis or after receiving antibiotic treatment [20]. To our knowledge, no studies have reported an association of *Enterococcus* spp. with *C. trachomatis* infection. However, *Enterococcus* (*Enterococcus faecalis* and *Enterococcus faecium*) have been isolated from vaginal specimens [20, 21].

*P. stutzeri* is a Gram-negative, aerobic, rod-shaped bacterium commonly found in water, soil, urine, blood, the respiratory tract, and surgical wounds [22]. The association of *P. stutzeri* with the vaginal microbiota has not been studied. A study characterizing the vaginal microbiota in Thai women found that *P. stutzeri*, *G. vaginalis*, and *A. vaginae* were frequently found in the non-lactobacilli dominated (NLD) group (23). Currently, no other studies have reported on the association of *P. stutzeri* with either *C. trachomatis* infection or the vaginal microbiota.

This study had several limitations. Firstly, only pregnant women living with HIV were included in this study. The physiological changes that occur during pregnancy may drive changes in the composition of the vaginal microbiota, resulting in a microbiome that is different from that of non-pregnant women. In addition, the gestational age of the participants may have confounded the microbiome analysis. Secondly, we did not collect data on antibiotic use. Therefore, women who were on antibiotic treatment at the time of sample collection may have been included in this study. Antibiotics have been postulated to interfere with a healthy vaginal microbiota, particularly causing a decrease in the abundance of *Lactobacillus* spp. [7]. Lastly, data on the treatment status and viral load of the participants was not collected. This might have confounded the microbiome analysis since antiretroviral therapy and a high viral load may influence the composition of the vaginal microbiota. Despite the small sample, this study provides evidence that there are differences in the vaginal microbiome of *C. trachomatis*-infected women compared to uninfected women. Future studies with larger cohorts will provide more clarity on the association between *C. trachomatis* infection and changes in the vaginal microbiota.

## Conclusion

The microorganisms that were significantly associated with *C. trachomatis* in this study cohort have not been previously observed in association with *C. trachomatis* infection or the vaginal microbiota. Therefore, future studies in larger cohorts that will investigate the role of these microorganisms in *C. trachomatis* infection and the vaginal microbiota are required. Furthermore, prospective studies that will investigate the cause-and-effect relationship between *C. trachomatis* infection and the vaginal microbiota are needed. These studies will provide clarity on whether *C. trachomatis* infection alters the vaginal microbiota or whether an altered vaginal microbiota increases susceptibility to *C. trachomatis* infection in women.

## Data Availability

The datasets used and/or analyzed during the current study are available from the corresponding author upon reasonable request.

## Abbreviations

BV: Bacterial vaginosis
HIV: Human immunodeficiency virus
PCR: Polymerase chain reaction
RCC: Rate of correct classification
STIs: Sexually transmitted infections
SCDFC: Standardized canonical discriminant function coefficient
